# Draft Genome Sequence of an Isolate of Colletotrichum fructicola, a Causal Agent of Mango Anthracnose

**DOI:** 10.1128/genomeA.00001-18

**Published:** 2018-02-22

**Authors:** Qili Li, Junyan Bu, Zhihe Yu, Lihua Tang, Suiping Huang, Tangxun Guo, Jianyou Mo, Tom Hsiang

**Affiliations:** a Institute of Plant Protection, Guangxi Academy of Agricultural Sciences, Nanning, Guangxi, China; b Guangxi Key Laboratory of Biology for Crop Diseases and Insect Pests, Nanning, Guangxi, China; c College of Life Science, Yangtze University, Jingzhou, Hubei, China; d School of Environmental Sciences, University of Guelph, Guelph, Ontario, Canada

## Abstract

Here, we present a draft genome sequence of isolate 15060 of Colletotrichum fructicola, a causal agent of mango anthracnose. The final assembly consists of 1,048 scaffolds totaling 56,493,063 bp (G+C content, 53.38%) and 15,180 predicted genes.

## GENOME ANNOUNCEMENT

Colletotrichum gloeosporioides *sensu lato* is a fungal species complex encompassing more than 20 closely related species, which interact with diverse herbaceous and woody plants as pathogens (1) or endophytes. Colletotrichum fructicola, a member of this species complex, is found on all five continents and is capable of causing severe diseases in a number of economically important plants, such as avocado, cocoa, pear, and tea oil trees (2), and has also been found to be one of the causal agents of mango anthracnose (3). The genome sequence of C. fructicola will help provide basic sequence information for studies on the biology, ecology, and evolution of this pathogen.

In this study, we present the draft genome sequence of C. fructicola strain 15060, which was isolated from infected mango leaves in Guangxi, China. The mycelium was grown on cellophane over potato dextrose agar (PDA), and 300 mg of mycelium was used for DNA extraction with the Qiagen DNeasy plant minikit (Qiagen, Mississauga, Canada). Genomic DNA (2 μg) was then sent, specifying 100-bp paired-end reads with a 300-bp insert, for sequencing at Genome Quebec (Montreal, Canada). Over 27.5 million paired-end reads totaling 6,854 Mb were received. The genome was assembled using the programs Velvet version 1.2.10 (4), ABySS version 2.0.1 (5), and SOAPdenovo version 2.04 with GapCloser version 1.12 (6), with odd-numbered kmers between 31 and 97. Assembly quality was assessed by examining the *N*_50_ value and the total number of scaffolds produced by the programs. The assembly with the highest *N*_50_ value was obtained using SOAPdenovo plus GapCloser at kmer 49, giving an *N*_50_ value of 480,736 bp and resulting in 1,918 scaffolds totaling 56,654,168 bp (G+C content, 53.37%). After scaffolds that were smaller than 200 bp were removed, the final assembly consisted of 1,048 scaffolds, with a genome size of 56,493,063 bp (G+C content, 53.38%) and an *N*_50_ value of 480,505 bp. Based on gene models from Colletotrichum gloeosporioides, a total of 15,180 protein-coding genes were predicted from this assembly using AUGUSTUS version 3.2.2 (7).

### Accession number(s).

This whole-genome shotgun project has been deposited in DDBJ/ENA/GenBank under the accession no. PHOC00000000. The version described in this paper is version PHOC01000000.
